# Inspection Score and Grading System for Food Services in Brazil: The Results of a Food Safety Strategy to Reduce the Risk of Foodborne Diseases during the 2014 FIFA World Cup

**DOI:** 10.3389/fmicb.2016.00614

**Published:** 2016-04-27

**Authors:** Diogo T. da Cunha, Ana L. de Freitas Saccol, Eduardo C. Tondo, Ana B. A. de Oliveira, Veronica C. Ginani, Carolina V. Araújo, Thalita A. S. Lima, Angela K. F. de Castro, Elke Stedefeldt

**Affiliations:** ^1^Faculdade de Ciências Aplicadas, Universidade de CampinasLimeira, Brazil; ^2^Curso de Nutrição, Centro Universitário FranciscanoSanta Maria, Brazil; ^3^Instituto de Ciência e Tecnologia dos Alimentos, Universidade Federal do Rio Grande do SulPorto Alegre, Brazil; ^4^Departamento de Nutrição, Universidade Federal do Rio Grande do SulPorto Alegre, Brazil; ^5^Departamento de Nutrição, Universidade Federal do Rio Grande do SulPorto Alegre, Brazil; ^6^Agencia Nacional de Vigilância SanitáriaBrasília, Brazil; ^7^Centro de Desenvolvimento do Ensino Superior em Saúde, Universidade Federal de São PauloSão Paulo, Brazil

**Keywords:** food safety, food inspection, risk assessment, public health surveillance, foodborne disease, food safety stamps

## Abstract

In 2014, Brazil hosted one of the most popular sport competitions in the world, the FIFA World Cup. Concerned about the intense migration of tourists, the Brazilian government decided to deploy a food safety strategy based on inspection scores and a grading system applied to food services. The present study aimed to evaluate the results of the food safety strategy deployed during the 2014 FIFA World Cup in Brazil. To assess food safety, an evaluation instrument was applied twice in 1927 food service establishments from 26 cities before the start of the competition. This instrument generated a food safety score for each establishment that ranged from 0.0 (no flaws observed) to 2565.95, with four possible grades: A (0.0–13.2); B (13.3–502.6); C (502.7–1152.2); and pending (more than 1152.3). Each food service received a stamp with the grade of the second evaluation. After the end of the World Cup, a study was conducted with different groups of the public to evaluate the acceptance of the strategy. To this end, 221 consumers, 998 food service owners or managers, 150 health surveillance auditors, and 27 health surveillance coordinators were enrolled. These participants completed a survey with positive and negative responses about the inspection score system through a 5-point Likert scale. A reduction in violation scores from 393.1 to 224.4 (*p* < 0.001) was observed between the first and second evaluation cycles. Of the food services evaluated, 38.7% received the A stamp, 41.4% the B stamp, and 13.9% the C stamp. All positive responses on “system reliability” presented a mean of 4.0 or more, indicating that the public believed this strategy is reliable for communicating risks and promoting food safety. The strategy showed positive results regarding food safety and public acceptance. The deployed strategy promoted improvements in the food safety of food services. The implementation of a permanent policy may be well accepted by the public and may greatly contribute to a reduction in foodborne diseases (FBDs).

## Introduction

Brazil has emerged on the global stage for sporting events, hosting major competitions such as the FIFA Confederations Cup and Robotic Cup in 2013, the FIFA World Cup in 2014, the World Indigenous Games in 2015, and the Olympic Games in 2016. According to data from the Brazilian Ministry of Tourism, Brazil received approximately one million foreign tourists of 203 nationalities during the 2014 FIFA World Cup. Additionally, three million Brazilian tourists traveled around the country during the competition (Brazil Tourism Ministry, 2014).

The FIFA World Cup can be classified as a mass-gathering event due the high number of people in a specific location for a specific purpose. A high concentration of people may increase the risk of transmission of emerging diseases and of outbreaks of food and waterborne diseases (Abubakar et al., 2012). As examples, foodborne disease (FBD) outbreaks caused by norovirus were documented in Germany during the World Cup of 2006 (Schenkel et al., 2006) and during the Olympic Summer Games in Greece in 2011 (Mellou et al., 2012).

Even though illnesses are not common events among tourists in Brazil, those that do occur are skin diseases, vector-borne fevers (e.g., Dengue and malaria), and some FBD (Wilson et al., 2014). In Brazil, the most common causes for the occurrence of these food outbreaks are the failure to control the time and temperature of ready-to-eat food, inadequate hygiene of the food handlers, equipment and utensils, and the use of raw food, in particular eggs with *Salmonella* spp. (Costalunga and Tondo, 2002; Lima et al., 2013).

Concerned about the intense migration of tourists for 2014 FIFA World Cup, the Brazilian government decided to make investments in areas involving public safety, tourism and health. The Brazilian government implemented actions on important issues such as epidemiological and environmental surveillance, controls in harbors, airports, and borders, public health emergencies, controls of events involving chemical, biological, radiological and nuclear hazards, worker health surveillance, sanitary surveillance focusing on foods and health, laboratories, attention to health, health promotion, communication, command, and control.

Considering the health surveillance of foods, the Brazilian government deployed a food safety strategy based on inspection scores and a grading system applied to food services. The strategy was developed to improve the food safety of food services within the destinations of 2014 FIFA World Cup and thus reduce the risk of FBD for Brazilians and tourists from other countries. This action was inspired by successful policies implemented in other cities and countries such as New York (City of New York - Department of Health and Mental Hygiene, 2007), Los Angeles (Buchholz et al., 2002), New South Wales (New South Wales - NSW Food Authority, 2011), Denmark (Denmark - Ministry of Food Agriculture and Fishing, 2012), and the United Kingdom (Food Standard Agency, 2012), where positive improvements were observed by their health departments.

These and most related food safety policies are the outcome of a complex trade-off between the interests of the different groups affected by the policy (Alphonce et al., 2014), including the professionals of health surveillance, food service managers, food service owners, retailers, and consumers.

In the food safety inspection score system, establishments are classified based on the health criteria set by health authorities. Food services receive a score based on their sanitary quality or alternatively on the degree of compliance with food safety regulations (Da Cunha et al., 2016). This classification is available to consumers, enabling them to know the food safety compliance of food services and to thus use this information to choose where to eat. The application of such systems has spread for its ability to improve the food safety of establishments by the awareness of citizens and the accountability of the health sector (e.g., health agencies, health ministry, and food departments) for ensuring compliance with sanitary regulations (Fielding et al., 2001).

To implement the Brazilian grading system, health surveillance officers of the Brazilian States and capital cities involved in the 2014 FIFA World Cup, food safety researchers and people responsible for the food retail sector discussed and organized the necessary actions to be taken in early 2012. It was decided by this group that the new strategy developed would be voluntary by the local authorities. Aiming to encourage adherence to this strategy, in May 2013 an ordinance was published (Brazil - Health Ministry, 2013) that outlined investing approximately U$2.0 mi. in the cities that adhered to the strategy. This resource was used to instrumentalize the local health surveillances and improve their working conditions.

After an extensive discussion, a risk-based checklist was developed to inspect the food services in the Brazilian capital cities participating in the 2014 FIFA World Cup. This inspection tool was based on current Brazilian regulations, focusing on the most important factors to be controlled to prevent FBD, and developed specifically for this strategy (Da Cunha et al., 2014). As results of the implementation of this strategy, 1927 food services were inspected in 26 Brazilian cities. The present study aimed to evaluate the results of the food safety strategy deployed during the 2014 FIFA World Cup in Brazil by analyzing the change of violation scores and public acceptance.

## Materials and methods

### Food services sample and strategy deployment

All steps of the study and strategy deployment were coordinated by the Brazilian Health Surveillance Agency (*Agência Nacional de Vigilância Sanitária—ANVISA* in Portuguese) with the involvement of the local authorities and partnerships with different Brazilian universities.

**Figure 1:**
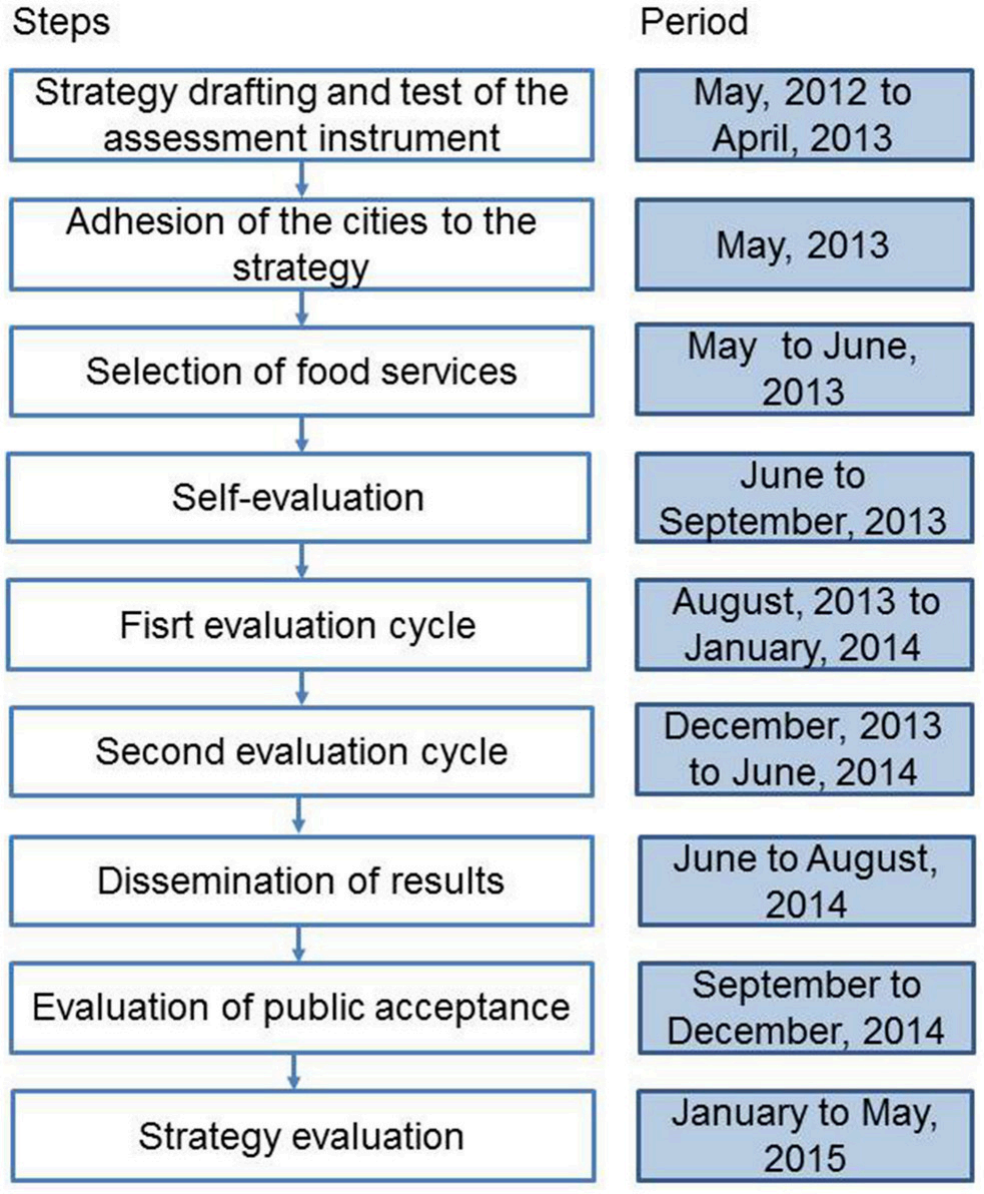
**Flow of the establishment and deployment of the food safety strategy based on inspection scores and the grading system for the Brazil 2014 World Cup**.

The strategy construction and consolidation occurred over 3 years from 2012 to 2015. Figure 1 shows the flow of the strategy deployment.

During the evaluation cycles, 1927 food services (bars, snack bars, and restaurants) were evaluated twice. From the 12 capital cities that hosted games as part of the 2014 FIFA World Cup, 11 acceded to the strategy, in addition to 15 cities near the capitals that did not host games but had a high influx of tourists. The number of establishments was proportional among the five regions of Brazil (South, Southeast, Midwest, North, and Northeast).

An intentional sample was used, including all food services inside the airports, the food services near the soccer stadiums and tourist spots, and restaurants considered as relevant by local health surveillance, due to the high influx of tourist and regional culinary consumers.

Health surveillance auditors and their coordinators were trained to standardize the evaluation process and minimize a possible evaluation bias. During the training, all items of the assessment instrument were presented and discussed. Additionally, the health surveillance auditors received a document with a detailed description of all items of the assessment instrument.

### Food safety assessment

The first step of the strategy was the development of a food safety assessment instrument. This instrument, a risk-based check-list with 51 items, was used to evaluate the food safety conditions and practices of food services. The check-list has nine categories: water supply; construction, facilities, equipment, furniture, and utensils; sanitization of the facilities, equipment, furniture, and utensils; integrated control of disease vectors and urban pests; handlers; raw materials, ingredients, and packaging; food preparation; storage and transport of the prepared food; responsibility, documentation, and record. Each of the 51 items received a specific score based on the risk of FBD. The checklist construction methods and reliability were described and discussed in a previously published paper (Da Cunha et al., 2014).

This instrument generated a food safety violation score for each establishment that ranged from 0.0 (no flaws observed) to 2565.95, with four possible grades: A (0.0–13.2); B (13.3–502.6); C (502.7–1152.2); pending (more than 1152.3). Each inspection item had a specific violation score deduction.

First, the owners and managers of the establishments received a guide about the strategy, its guidelines and methods (Agencia Nacional de Vigilância Sanitária, 2013). They were encouraged to conduct a self-assessment to become familiar with the requirements and to be prepared for the assessment by the Health Surveillance. Two to six months after the self-evaluation, the food services were evaluated by a Health Surveillance auditor. The evaluation instrument was applied over two evaluation cycles. A minimum interval of 4 months between assessments was established.

All evaluations were performed without prior notice to the food service's owner or manager. During the evaluation the Health Surveillance auditor observed the practices, procedures, physical aspects, and documents present in the food service. All the check-list items were answered with one of three possible answers: yes, no or not applicable. The evaluations took from 45 min to 4 h, depending on the size and complexity of the establishment.

The data of the food safety evaluation were inserted by the Health auditors in an online system, specially developed to tabulate the data and generate individual or general reports.

The first evaluation cycle occurred during August 2013 to January 2014, and the second evaluation cycle occurred during December 2013 to June 2014. The overlap of months between the two evaluation cycles occurred because the States started the first evaluation cycle in different periods.

**Figure 2:**
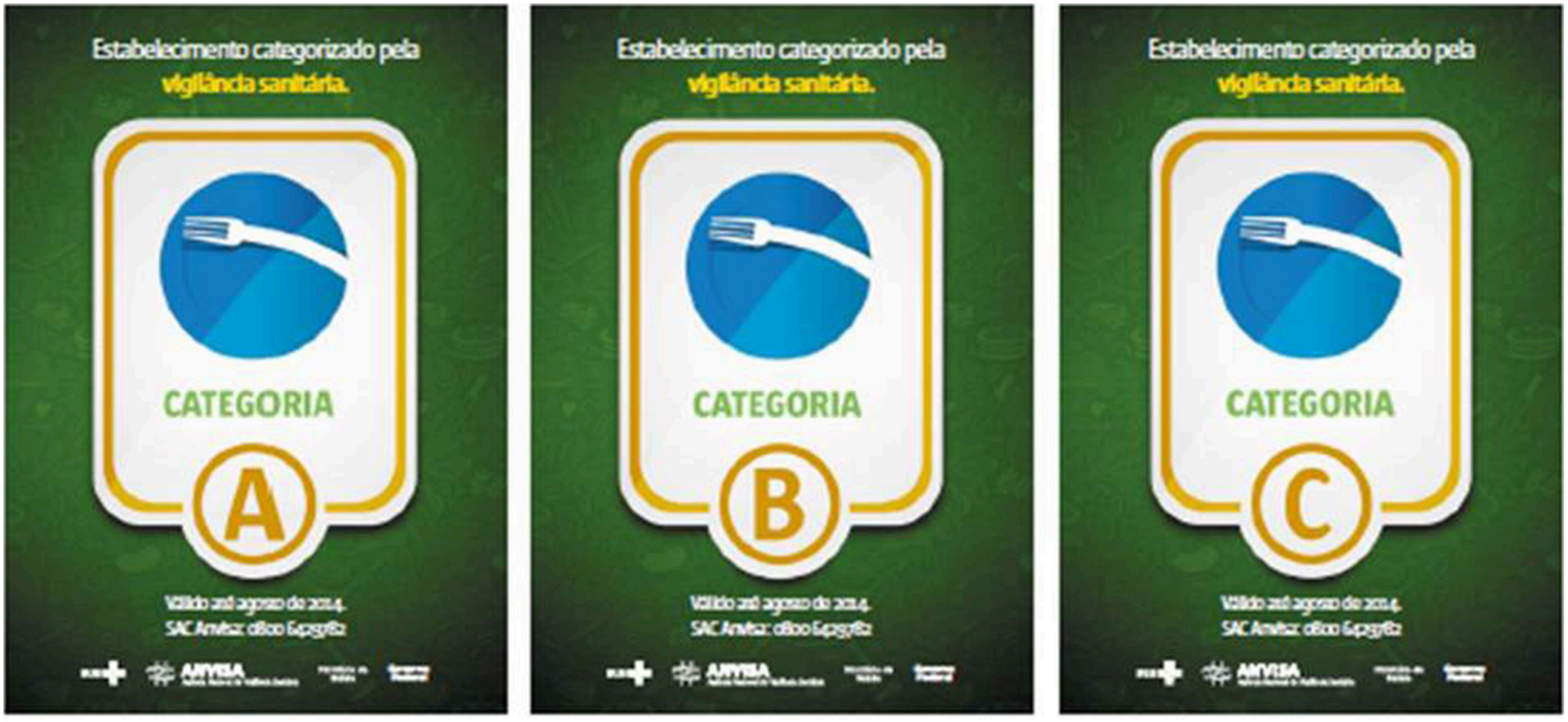
**Food safety stamps used during the Brazil 2014 World Cup**.

During the first cycle, the Health Surveillance auditors made the observed violations known to the owners and managers. This step was used to educate and enable the food services to correct the flaws and violations prior to the second cycle. The second cycle defined the grade (A, B, C, or pending) for the establishments after the second evaluation (Figure 2). Establishments with a pending grade did not receive a grade stamp but were encouraged by Health Surveillance to improve their practices.

### Public acceptance

After the World Cup's end, a study was conducted with different groups of the public to evaluate the acceptance of the strategy. In total, 1411 individual evaluations were performed by 221 consumers, 998 food service owners or managers, 150 Health Surveillance auditors, and 27 Health Surveillance coordinators. These participants completed a survey with positive and negative responses about the inspection score system. Service owners or managers completed the forms during a visit for health auditors after the World Cup. Consumers, Health Surveillance auditors and coordinators completed an online survey. These responses were organized into three categories: system reliability, practical applications, and strategy continuity. The answers were given through a 5-point Likert scale (1—strongly disagree to 5—strongly agree) (Likert et al., 1993).

To assess the consumers' perception about the grade stamps, they were asked to give their opinion about what represented an establishment with A, B, and C stamps. The answers were given through a 5-point scale (1—very good to 5—very poor). A sixth option of “I do not know” was included to minimize an answer bias.

### Statistical analyses

The variables were expressed as the mean and the standard deviation. Violation scores between the two evaluation moments were compared using a paired Student's *t*-test since all variables presented adherence to the normal curve and homoscedasticity. To compare the change in dichotomous variables between the two cycles, McNemar's test was used. Maps were plotted to show the evolution of inspection scores during the cycles among the Brazilian States.

For the data analysis, the software SPSS 15.0 was used. In all tests *p* < 0.05 was considered significant.

## Results

A reduction in violation scores between the first and the second evaluation cycles was observed. At first, the food services presented a mean (standard deviation) violation score of 393.1 (432.7), followed by a mean score of 224.4 (352.0) in the second cycle (*p* < 0.001).

Table 1 shows the classification of establishments and their evolution between assessments. Few establishments showed a reduction in their classification. A total of 69 establishments were classified as A in the first evaluation and passed to B in the second, and five were classified as A and passed to C. In contrast, a high number of establishments had improved their rating. Among the establishments classified as B in the first cycle, 339 were classified as A in the second cycle of evaluation. For the establishments classified as C in the first cycle, 93 went to A and 211 to B.

**Table 1:** **Grade changes between the two evaluation cycles of the evaluated food establishments (Brazil, 2014)**.

		**Second evaluation cycle (Final grade)**				**Total (%)**
		**A**	**B**	**C**	**Pending**	
First evaluation cycle	A	289	69	5	1	358 (18.8)
	B	339	410	66	10	823 (42.8)
	C	93	211	114	33	447 (23.4)
	Pending	25	108	84	70	284 (14.8)
	Total (%)	746 (38.7)	798 (41.4)	269 (13.9)	114 (5.9)	1927 (100%)

After the second evaluation cycle, 38.7% of the food services achieved a classification with a better score and were classified as A, 41.4% as B, and 13.9% as C.

**Figure 3:**
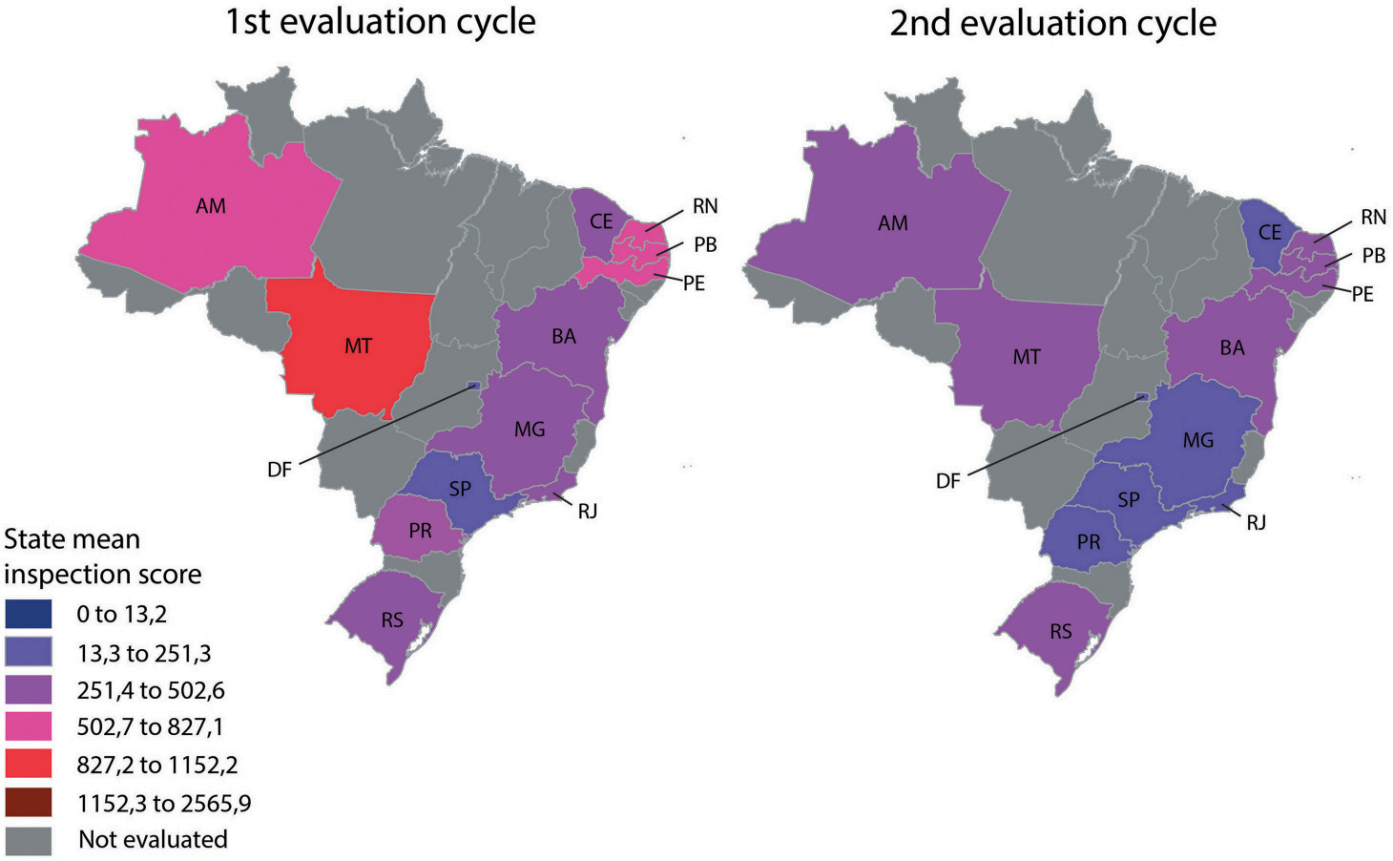
**Brazil map showing the evolution of inspection scores between the two evaluation cycles in each State**. Number of evaluated establishments per state (capital, airport, and tourist cities): AM, capital (118); MT, capital (78), airport (4); DF, capital (212), airport (19); RS, capital (77), airport (18), two tourist cities (87); PR, Capital (171), airport (13); SP, capital (282), airport (43), one tourist city (29); RJ, capital (243), airport (21), one tourist city (39); MG, capital (97), airport (1); BA, airport (19), one tourist city (13); PE, capital (158), airport (18), four tourist cities (81); PB, capital (57); RN, capital (130), airport (9), three tourist cities (47); CE, capital (75), airport (13).

The violation score (mean) was reduced between the assessments. Figure 3 shows a Brazil map indicating the inspection score reduction by considering the mean value presented by food services in each Brazilian State. In general, the States in the South and Southeast had food services with lower scores in the first cycle than the food services from the States in the North, Northeast, and Midwest. However, a more homogenous average score was observed in all States in the second evaluation cycle.

When evaluated individually, 49 of the 51 violations of the assessment instrument showed a positive evolution (*p* < 0.001) between the two cycles when McNemar's test was used. The two items that did not show a positive evolution were “facilities with running water” and “facilities with connections to the sewer system or septic tank.” However, it is noteworthy that these items were mandatory.

The violation percentage of all evaluated categories was reduced significantly between the evaluation cycles (Table 2). It is important to note that any foodborne outbreak involving the evaluated food services was notified during the World Cup.

**Table 2:** **Differences in the violation percentage of each category of the evaluation instrument between the two evaluation cycles (*n* = 1927; Brazil, 2014)**.

**Categories**	**Violation percentage**		***p***
	**1st evaluation cycle**	**2nd evaluation cycle**	
Water supply	6.1	3.2	<0.001
Construction, facilities, equipment, furniture, and utensils	21.3	13.4	<0.001
Sanitization of the facilities, equipment, furniture, and utensils	17.1	11.3	<0.001
Integrated control of disease vectors and urban pests	19.5	12.1	<0.001
Handlers	11.6	5.9	<0.001
Raw materials, ingredients, and packaging	18.8	12.2	<0.001
Food preparation	23.2	13.9	<0.001
Storage and transport of the prepared food	20.4	12.2	<0.001
Responsibility, documentation, and record	36.9	24.1	<0.001

The “responsibility, documentation and record” was the category with the greatest reduction in the violation percentage, and the “water supply” category had the lower violation percentage.

Table 3 shows the public acceptance of the scoring system. All positive responses from “system reliability” showed a mean of 4.0 or more, indicating that public believed that this strategy was reliable for communicating risks and to promoting food safety.

**Table 3:** **Public (consumers, food service owners and managers, health surveillance coordinators and auditors) acceptance of the inspection score and grading system deployed during the 2014 FIFA World Cup in Brazil (Brazil, 2014)**.

**Responses**	**Consumers *n* = 221 Mean ± SD^†^**	**Food service owners or managers *n* = 998 Mean ± SD^†^**	**Health Surveillance auditors *n* = 150 Mean ± SD^†^**	**Health Surveillance coordinators *n* = 27 Mean ± SD^†^**
**CATEGORY: SYSTEM RELIABILITY**				
1 Inspection score and grading systems may increase the credibility of food services	4.6 ± 0.7	4.7 ± 0.5	4.4 ± 0.8	4.3 ± 0.9
2 ^*^Inspection score and grading system have little influence on the quality of food	2.5 ± 1.5	2.3 ± 1.5	2.2 ± 1.4	1.9 ± 1.3
3 Inspection score and grading system is a good strategy to inform consumers about the quality of food	4.5 ± 0.7	4.7 ± 0.6	4.4 ± 0.9	4.2 ± 1.1
4 Inspection score and grading system increase consumer confidence of Health Surveillance	4.4 ± 0.8	4.8 ± 0.5	4.3 ± 1.0	4.0 ± 1.1
5 ^*^Inspection score and grading system is unnecessary	1.6 ± 1.0	1.7 ± 1.3	1.8 ± 1.1	1.9 ± 1.4
6 Inspection score and grading system promoted improvements in the food safety of food services	Na	4.6 ± 0.7	4.4 ± 0.8	4.3 ± 0.8
7 Inspection score and grading system promotes improvements in my food service	Na	4.6 ± 0.7	Na	Na
8 Inspection score and grading system enhances the food services that invest in the implementation of good handling practices	Na	4.8 ± 0.5	4.6 ± 0.9	4.5 ± 0.8
**CATEGORY: PRACTICAL APPLICATIONS**				
9 ^*^The use of alphabetical letters (A, B, and C) is an inadequate form to communicate the grading to the consumers	Na	2.3 ± 1.5	2.9 ± 1.5	3.0 ± 1.5
10 Your clients (You^‡^) have used the category A, B or C as criteria for choosing your establishment	4.3	4.0 ± 1.0	Na	Na
11 I had difficulty understanding the stages of the grading system	Na	2.2 ± 1.4	Na	Na
12 The checklist has the necessary information to evaluate the good handling practices	Na	4.6 ± 0.7	3.9 ± 1.1	3.9 ± 1.0
13 The proposed checklist helps to identify the risks of food services	Na	4.7 ± 0.6	4.2 ± 1.0	4.0 ± 1.1
14 ^*^The grade system is not the role of Health Surveillance	Na	1.8 ± 1.3	1.8 ± 1.4	2.3 ± 1.6
15 ^*^It is difficult to integrate the proposed checklist in my professional practice	Na	Na	2.5 ± 1.5	2.1 ± 1.4
16 The checklist shall also apply to restaurants, bars and snack bars	Na	Na	3.3 ± 1.4	3.2 ± 1.4
**CATEGORY: STRATEGY CONTINUITY**				
17 The score and grading system has improved my perception about the Health Surveillance supervision	Na	4.5 ± 0.8	4.2 ± 1.1	4.1 ± 1.1
18 The score and grading system brought improvements in the Health Surveillance work process	Na	Na	3.6 ± 1.3	3.8 ± 1.3
19 ^*^The grading system overloads the Health Surveillance services	Na	Na	3.4 ± 1.5	3.7 ± 1.4
20 The grading system should continue after the 2014 FIFA World Cup	Na	4.6 ± 0.8	4.2 ± 1.3	4.0 ± 1.6

* Responses with a negative reference; SD, standard deviation; na, not asked.

† 1, strongly disagree; 2, disagree; 3, neutral; 4, agree; 5, strongly agree.

‡ Question adjusted for the consumers.

Regarding practical applications, professionals from Health surveillance believed that the proposed instrument helped them to identify risks but should have included more information to help them to assess food safety (response 12) and that the instrument was not equally applied to all food businesses (response 14).

Health surveillance auditors and coordinators demonstrated a neutral perception, with a tendency to agree that this system burdened the Health Surveillance services. However, along with the food services owners and managers, they believed that the inspection score and grading system must continue after the World Cup and be applied in all of Brazil.

Finally, consumers were asked their opinion about the stamps used (Table 4). Most (79.6%) believed an establishment with an A stamp was a very good one. In relation to the B and C stamps, most of the consumers evaluated the establishment as a good one and a regular one, respectively. An important percentage of consumers (45.2%) believed that an establishment with a C stamp was poor or very poor in regards to food safety.

**Table 4:** **Consumers' perception about the stamps used to grade food services during the 2014 FIFA World Cup in Brazil, (2014)**.

**Ascertain**	**Consumers' opinion (in percent) *n* = 221**					
	**Very good**	**Good**	**Regular**	**Poor**	**Very poor**	**I do not know**
A food service that received the A stamp, in my opinion, is a …establishment:	79.6	14.5	2.3	0.5	0.5	2.7
A food service that received the B stamp, in my opinion, is a …establishment:	5.0	53.0	32.9	2.7	0.5	5.9
A food service that received the C stamp, in my opinion, is a …establishment:	1.8	4.1	42.5	30.1	15.1	6.4

## Discussion

The creation and deployment of a food safety strategy based on an inspection score and grading system was prompted by three premises: (a) inspection scores may successfully predict foodborne outbreaks, as observed from experiences in other places (Irwin et al., 1989; Buchholz et al., 2002; Zablotsky Kufel et al., 2011); (b) they may be able to communicate risks effectively to the consumer in honest and unbiased way, which is a duty of the government; and (c) an inspection score and grading system may promote a “healthy competition” among food service owners and managers. To achieve a better grade or a grade better than their concurrent one, owners and managers must invest in food safety issues, consequently reducing the risk of an FBD. This positive effect of the grading system was observed in Los Angeles in the United States (Fielding et al., 2001).

As expected, the food service managers and owners invested in food safety during the strategy deployment. This investment improved food safety, as observed by the significant reduction in inspection scores between the two evaluation cycles (393.1–224.4 on average). Most of inspection items demonstrated a positive evolution with the exception of the ones regarding access to running water and connection to a sewer system, which already showed a high compliance percentage in the first cycle. The costs of basic food safety requirements (e.g., correct food handler practices and behavior, application of an effective control and monitoring system, and performance of adequate sanitization of equipment) can be reasonable (Mortlock et al., 2000; Lockis et al., 2011) and have a high cost-benefit for the government (Crutchfield et al., 1997).

In a recent study in United States, it was observed that consumers, mainly women, are willing to pay more for their meal for increased food safety (Alphonce et al., 2014). In addition, in Brazil the consumption of food away from home rose from 22.2% in 2002–2003 to 27.9% in 2008–2009, expanding and strengthening the retail food sector (Claro et al., 2014). These data promote insights that investments in food safety could be used as a marketing strategy to entice more consumers in this expanding scenario.

Another positive effect of the strategy deployment was the reduction in key violations associated with FBD. Studies highlight that inadequate temperatures to hold food (hot and cold ones) and general hygiene (food handler, equipment, and utensil hygiene) are the main causes of foodborne outbreaks (Todd et al., 2007; ESR, 2008; Norrung and Buncic, 2008; Food and Drug Administration, 2009, including in Brazil (Costalunga and Tondo, 2002; Lima et al., 2013). During the second evaluation cycle, food services were more likely to hold food at safe temperatures, had improved food handler hygiene (especially hand hygiene), included a food protection supervisor and applied control methods (i.e., supervision, documentation, and records). Similar results were observed in New York in the United States 18 months after the grade system deployment (City of New York - Departament of Health and Mental Hygiene, 2012).

Based on official epidemiological data (Brasil, 2011), most foodborne outbreaks in Brazil have occurred inside private homes, where Health Surveillance have no action. The second place with higher occurrence of foodborne outbreaks were restaurants, and this was one of the reasons why classification and inspections were conducted in such food services. Furthermore, the strategy was planned to control the most important pathogenic microorganisms in Brazil (i.e., *Salmonella, S. aureus, E. coli*, and *B. cereus*). The items of the check-list were designed in order to control these microorganisms reducing the risk of foodborne outbreaks (Da Cunha et al., 2014). Once the strategy has been implemented for a short period of time (during the 2014 World Cup) and involved a specific number of food service, it has not been possible to observe impact on the overall cases of foodborne outbreaks yet. It will be probably be possible to observe a reduction in FBD after the conversion of the strategy into a permanent policy, as noted in New York City (City of New York - Departament of Health and Mental Hygiene, 2012).

None foodborne outbreaks involving the evaluated food services were notified during the World Cup. However, it is not possible to establish a cause-effect relationship between the strategy and outbreaks. In another study the authors stated that the restaurant grade does not correlate with microbiological quality of the food (Kjeldgaard et al., 2010). Many aspects can influence the correlation of outbreak data and inspection data (e.g., FBD sub-notification, underdiagnosis, problems with laboratory testing, and variation in seeking medical care; Scallan et al., 2011). Additionally, the strategy was deployed to reduce the risk by reducing food safety violations and controlling important food safety procedures. It is not possible to infer that a food service with higher violation scores might cause a foodborne outbreak, but this food service could offer a meal with higher likelihood of contamination, that can cause FBD or not (Da Cunha et al., 2016).

The food safety strategy based on inspection scores and a grading system was established to reduce the risk of FBD during the World Cup in Brazil, but other important improvements could be observed. It was the first time that a grade system was implemented by the government to improve the food safety, which was new for auditors, researchers, and consumers. Each Brazilian city has its own method to assess food services. Thus, this strategy also promoted the creation of a standardized and reliable assessment instrument for health surveillance, with a transparent assessment so the owners of food services and consumers had access to this instrument and the grade system methodology.

Being a temporary system with an expected end date, no strategy was designed to show the history of past assessments of the food services. Grading systems from other countries used this strategy to encourage food services owners to keep investing in food safety and not just prior to a food safety assessment, as recommended by Buchholz et al. (2002) and used in the Denmark grading system (Denmark - Ministry of Food Agriculture and Fishing, 2012). Thus, if the Brazilian government implements the system permanently, it is recommended that the implementation of strategies includes food services' historic violations. As the city of Rio de Janeiro was one of the places where the strategy was deployed, the positive effects achieved are possibly lasting and thus the risk of FBD during the Olympic Games in 2016 will be reduced. However, it is suggested that the local Health Surveillance make the revaluation of the establishments in Rio de Janeiro prior to Olympic Games.

The scoring system, effective in reducing the risk of FBD, was also intended to be a communication strategy with the consumer and also a strategy to facilitate the work of Health Surveillance in Brazil. Considering these issues, the perceptions of consumers, owners of establishments, and representatives of Health Surveillance on this scoring system were evaluated. All interviewed groups agreed that the inspection system may increase the credibility of the food services, may influence the quality of food, is a good strategy for communicating with consumers and may increase the consumers' confidence in Health Surveillance. More importantly, most of these participants disagreed that the scoring system was unnecessary. These data are important as they may provide impetus to the government for investment in the inspection system. However, stakeholders must be aware that the consumers generally tend to feel completely safe about consuming food away from home because they believe that regulations may protect them (Wilcock et al., 2004). The grading system may strengthen this feeling, mitigating consumer protection attitudes in these food services. These data reinforce the importance of proper communication. The government must state to consumers that the scoring system may improve food safety, but is impossible to ensure absolute safety, even for “A” stamp food services.

Respondents raised concerns regarding use the letters used as a communication strategy, especially Health Surveillance coordinators. Letters are a common symbol used in food safety score systems, which are used in New York (City of New York - Departament of Health and Mental Hygiene, 2010), Los Angeles county (Fielding et al., 2001), San Diego county (Filion and Powell, 2009), and New South Wales (New South Wales - NSW Food Authority, 2011). The study conducted by the New South Wales Food Authority (New South Wales - NSW Food Authority, 2011) identified that consumers may not understand that grades such as B and C are also acceptable. This was partially observed in our study, as consumers defined a “B” establishment as good or regular ones and defined a “C” establishment as regular or poor.

A trusted system, which communicates food risks efficiently and is accepted by the society, must be based in competence and honesty (Frewer, 2000). This is likely why people disagree that a grading system is not a duty of health inspection. Trust in food risk communication is based on the knowledge, accuracy and public welfare interest of the communicator (Frewer et al., 1996), features that should be natural for government authorities. Furthermore, a private organization would charge food services to grade them, creating a cycle in which only establishments with greater resources would benefit from the “A” stamp.

Apparently, all interviewed groups agreed that the scoring system should continue after the World Cup. Additionally, the strategy construction and deployment initiated a dialogue between the food service sector and Health Surveillance with the participation of universities. The involvement of the government with the private sector and educational institutions may promote a democratic and fruitful discussion on the design and implementation of public policies. It is suggested that the scoring system should be used in other mass gathering events such as the 2016 Olympic Games in Rio de Janeiro.

## Conclusion

The food safety strategy based on an inspection score and grading system, deployed before and during the 2014 FIFA World Cup in Brazil, showed positive results regarding food safety and public acceptance. The deployed strategy promoted improvements in the food safety of food services.

Based on the results, it is believed that a score system policy has great potential to:

- reduce the risk of FBD in Brazil;
- motivate the food service owners to invest in food safety;
- communicate risks to the consumer in a simple way;
- be acceptable to the public and stakeholders;
- facilitate inspections, focusing on control of the most important issues to be controlled to prevent FBD.

The implementation of a permanent policy may be well accepted by the public and may greatly contribute to the reduction in FBD. The discussion about turning this strategy into a policy should be initiated. Moreover, an inspection score and grading system for food services conducted by government authorities may inspire trust.

## Author contributions

All authors listed, have made substantial, direct and intellectual contribution to the work, and approved it for publication.

## Funding

Part of the publication fees were supplied by FUNCAMP - Fundação de Desenvolvimento da Unicamp (Grant n.2169/16).

### Conflict of interest statement

The authors declare that the research was conducted in the absence of any commercial or financial relationships that could be construed as a potential conflict of interest.
